# Doctoral students’ well-being: a literature review

**DOI:** 10.1080/17482631.2018.1508171

**Published:** 2018-08-13

**Authors:** Manuela Schmidt, Erika Hansson

**Affiliations:** a Faculty of Health Science, Kristianstad University, Kristianstad, Sweden; b Department of Health Science, Lund University, Lund, Sweden; c Faculty of Education, Kristianstad University, Kristianstad, Sweden

**Keywords:** Doctoral student, PhD student, well-being, review, SWOT

## Abstract

**Purpose:** Doctoral student well-being is an important matter that shapes the well-being of academics throughout their careers. Given that well-being has been found to be closely related to employee productivity and efficiency, strategies associated with maintaining well-being during PhD studies might be crucial for higher education, its outcomes and—just as importantly—for a balanced life of PhD students.

**Method:** Based on 17 studies, this literature review critically assesses the literature on doctoral student well-being.

**Results:** Theoretical models, concepts of well-being, and methods applied are discussed, as are the results of the articles. The reviewed studies are then discussed based on a SWOT analysis addressing the strengths and weaknesses of the reviewed research as well as the identified opportunities and threats, which can be used as a basis for future research. Based on the review findings and the SWOT analysis, a multidimensional view of the well-being of doctoral students is proposed.

**Conclusions:** The study proposes a more student-centred approach to meeting doctoral students’ needs, and the enhancement of doctoral student well-being in order, as a long-term goal, to improve academics’ well-being and productivity.

## Introduction

Several studies suggest that academic staff develop strategies to maintain and enhance their well-being early in their academic careers (cf. Agevall, Broberg, & Umans, 2016; cf. Lease, 1999; Petersen, 2011; Salmela-Aro, Tolvanen, & Nurmi, 2011). These early years, i.e., the years spent on PhD studies, are usually associated with a transition from dependence to independence, i.e., from the student role to the professional academic role (Laudel & Gläser, 2008), and it is in the intersection of this transition, and its associated decisions and uncertainties, the future well-being of aspiring academics possibly develops (Schmidt & Umans, 2014; Stubb, Pyhältö, & Lonka, 2011).

Understanding the well-being of individuals in the work setting—where they spend most of their adult life in (Greenberg et al., 2003)—is an emancipating endeavour to pursue (cf. Liu, Siu, & Shi, 2010). Usually, individuals’ well-being in work settings is closely related to organizational functioning. Being a key resource in higher education institutions (HEIs), academic staff, including doctoral students, play a major role in achieving the objectives of higher education and their performance affects student learning and success (de Lourdes Machado, Soares, Brites, Ferreira, & Gouveia, 2011), significantly influencing the success in any educational programme (Stankovska, Angelkoska, Osmani, & Grncarovska, 2017). However, academic staff have been identified over the years as the occupational group in HEIs that experience the most volatile well-being at work (e.g., Abouserie, 1996; Craig, Hancock, & Craig, 1996; Taris, Schreurs, & Van Iersel-Van Silfhout, 2001). Research investigating the well-being of academics is fragmented as well as limited when it comes to explaining the particular factors that contribute to this volatility (Kinman, 2008). Understanding the precursors of well-being in this occupational group is important given that the well-being of academics might affect their productivity in both research and teaching, ultimately influencing the quality of higher education (Vera, Salanova, & Martin, 2010). Poor well-being among those remaining in academia could be detrimental to their engagement in research and teaching, and might also imprint on the doctoral students they will supervise in the future. The well-being of this occupational group also has both short- and long-term consequences and might be an important enabler not only of educational quality but also of the sustainability of education systems. However, this calls for that the doctoral students, at the beginning of their career, are given the right tools to remain healthy in their work environment. Doctoral studies are often characterized by constant peer pressure, frequent evaluations, low status, high workload, paper deadlines, financial difficulties, pressure to publish, active participation in the scholarly environment, including conferences (Kurtz-Costes, Helmke, & Ulku-Steiner, 2006; Maysa & Smith, 2009), lack of permanent employment, and an uncertain future (Huisman, de Weert, & Bartelse, 2002). Feelings of uncertainty and poor relationships with supervisors (Lovitts, 2001) are additional stressors, as are the numerous roles doctoral student are expected to take, e.g., as a student, employee, parent, or researcher (Martinez, Ordu, Della Sala, & McFarlane, 2013; Schmidt & Umans, 2014). In light of the number of potential stressors and the complex work situation of doctoral students, it is a challenge for them to maintain a healthy work–life balance (Golde, 2005). Attrition rates are high, up to 50%, depending on doctoral discipline and country (Gardner, 2008; Jiranek, 2010; Lovitts & Nelson, 2000) and some leave academia after completing their doctoral programme, pursuing other careers. Furthermore, there is evidence that the scholarly communities do not always provide optimal opportunities for doctoral students to participate in. Instead, the milieu is perceived as burdensome by a number of doctoral students, which affects their well-being in a negative way (Stubb et al., 2011). There are also indications that doctoral students (especially women) suffer from stress and mental fatigue (Appel & Dahlgren, 2003).

Previous studies of the well-being of doctoral students, and of academic staff in general, have primarily concentrated on isolated determinants of well-being instead of taking a multidimensional perspective, which would allow consideration of multiple factors that interact with each other in simultaneously shaping well-being (Moberg, 1979). By reviewing the literature, this study aims to critically and systematically assess previous research on doctoral student well-being and give suggestions for future research by performing a SWOT analysis.

### The concept of well-being

Well-being is a multifaceted phenomenon that has been studied in a number of different disciplines and thus has been defined in many different ways. Either due to or despite the multiplicity of defini-tions it has been described as a *“catch-all category”* (Cameron, Mathers, & Parry, 2006, p. 347) that is still lacking an overall accepted definition (Seedhouse, 1995).

It is common to use “health” as a starting point in defining well-being, probably due to the World Health Organization which included well-being in its definition of health by declaring that *“Health is a state of complete physical, mental and social well-being and not merely the absence of disease or infirmity”* (WHO, 1948, p. 100). However, the definition of health is also rather problematic as it uses the unclear concept “well-being” in its definition, and has occasionally been criticized as utopian (Larson, 1999). Another take on well-being was presented by Galvin and Todres in their conceptual framework consisting of “the Dwelling-mobility lattice’’ (Galvin & Todres, 2011) stating that well-being—independent of health and illness—can be experienced spatially, temporally, inter-personally, bodily, in mood and in terms of the experience of personal identity. They state that well-being is more complex than health and is not limited to any setting or role, e.g., work place well-being or the role of being a student, thus their definition focuses on the essence of well-being (Galvin & Todres, 2011; Todres & Galvin, 2010). In the last decades two dominating perspectives of well-being have emerged: psychological (or eudaimonic) well-being that is concerned with the realization of a person’s true nature and potential; and subjective (or hedonic) well-being that is based on the general idea that happiness and pleasure form the essential goal of human life (Diener, 2018; Ryan & Deci, 2001; Ryff & Keyes, 1995). Both perspectives are relatively distinct and originate from different philosophical views, yet they overlap (Lundqvist, 2011).

While acknowledging the debate and the criticism on the definitions of well-being and health, the focus of this review is doctoral students’ well-being, and there is no intention to present yet another definition of the concept. Therefore, the starting point has been Medin and Alexanderson (2001), definition, which describes well-being as “*the individual’s experience of his or her health*” (p. 75). This comprehensive view of well-being, which highlights the individuals’ constantly changing experiences, is used as a framework for this study. In addition, the multidimensional and pragmatic approach to well-being presented by Ryff (1989) has been used as guidance, stressing the importance of positive relationships with others, personal growth, environmental autonomy, autonomy, purpose in life, as well as self-acceptance to maintain well-being.

## Method

### Systematic literature review

For the purpose of this review, a systematic literature search was conducted in March 2018 of the following databases: Web of Science (all databases), ERIC, PsycInfo, and Education Research Complete. The search included the following keywords: well-being OR wellbeing OR “well being” AND “doctoral student*” OR “phd student*” OR “doctoral graduate*” as shown in Table I. The search was limited to the keywords in the abstracts (or topic in Web of Science) and no time limitations were chosen.

**Table I.** Search process and items found.

		Databases				
Order of search	Search action	PsycINFO	Web of Science (all databases)	ERIC	Education Research Complete	Total number of articles
1.	AB wellbeing OR AB well-being OR AB “well being”	72.734	130.733^a^	11.057	17.891	232.415
2.	AB “doctoral student*” OR AB “phd student*” OR AB “doctoral graduate*”	2.349	3.546^a^	2.316	2.679	10.890
3.	1 AND 2	50	29	10	19	108
4.	Filter: English language (- 6)	48	26	9	19	102
5.	Filter: peer-reviewed (- 34)	22	22^b^	7	17	68
6.	Reduction of duplicates (−20)					48
7.	Reduction by lack of relevance (−31)					17

^a^Search “Topic” which included title, abstract and keywords.

^b^Document type: article.

### Inclusion criteria and selection process

The eligibility criteria for the publications to be reviewed were: (1) inclusion of an empirical investigation, (2) specific focus on doctoral students and their well-being, (3) peer-reviewed and published in a scientific journal, (4) and written in English. Articles referring to postgraduate students were thoroughly examined because the term “postgraduate” refers to doctoral students in some educational systems but not in others. If it was clear that the author(s) were referring to master’s students, the articles were excluded.

## Findings

In total, 68 articles were identified in the databases selected, as shown in Table I. After screening the abstracts/articles for relevance and excluding duplicates, 17 articles remained to be included in the literature review, which are presented in Table II. The excluded articles did not satisfy the inclusion criteria, i.e., though they were related (e.g., concerned with coping, resilience, or group writing) they had too little focus on well-being, or involved doctoral students only as a minority in the data collection.

**Table II.** Summary of the literature review.

Authors/year	Title	Country	Sample	Design
1. Pychyl and Little (1998)	Dimensional specificity in the prediction of subjective well-being: Personal projects in pursuit of the PhD	Canada	I: 19 PhD students in interviews, II: 81 in survey	Qualitative/ quantitative
2. Stubb et al. (2011)	Balancing between inspiration and exhaustion: PhD students’ experienced socio-psychological well-being	Finland	669 PhD students	Qualitative/quantitative
3. Haynes et al. (2012)	My world is not my doctoral programme…or is it?: Female students’ perceptions of well-being	USA	8 PhD students	Qualitative
4. Juniper et al. (2012)	A new approach to evaluating the well-being of PhD research students	UK	57 PhD students in interviews (34 in focus groups)/1202 in survey	Qualitative/quantitative
5. Pyhältö and Keskinen (2012)	Doctoral students’ sense of relational agency in their scholarly communities	Finland	669 PhD students	Qualitative/ Quantitative
6. Stubb et al. (2012)	The experienced meaning of working with a PhD thesis	Finland	669 PhD students	Qualitative/ quantitative
7. Martinez et al. (2013)	Striving to obtain a school-work-life balance: the full-time doctoral student	USA	5 PhD students	Qualitative
8. Caesens et al. (2014)	The impact of work engagement and workaholism on well-being	Belgium	343 PhD students	Quantitative
9. Schmidt and Umans (2014)	Experiences of well-being among female doctoral students in Sweden	Sweden	12 PhD students	Qualitative
10. Shavers and Moore (2014)	Black female voices: Self-presentation strategies in doctoral programmes at predominately white institutions	USA	15 PhD students	Qualitative
11. Anttila et al. (2015)	The added value of a PhD in medicine—PhD students’ perceptions of acquired competences	Finland	163 PhD students	Qualitative/ Quantitative
12. Hunter and Devine (2016)	Doctoral students’ emotional exhaustion and intentions to leave academia	Nine countries (most participants from Canada and the USA)	186 current or recently graduated PhD students	Qualitative/quantitative
13. Cornér et al. (2017)	The relationships between doctoral students’ perceptions of supervision and burnout	Finland	248 PhD students	Quantitative
14. Herrmann and Wichmann-Hansen (2017)	Validation of the quality in PhD processes questionnaire	Denmark	1670 PhD students	Quantitative
15. Ziapour et al. (2017)	Prediction of the dimensions of the spiritual well-being of students at Kermanshah University of medical sciences, Iran: the roles of demographic variables	Iran	346 PhD students	Quantitative
16. Zahniser, E., Rupert, P.A., & Dorociak, K.E. (2017)	Self-care in clinical psychology graduate training	USA	358 PhD students	Qualitative/ Quantitative
17. Kumar and Cavallaro (2018)	Researcher Self-Care in emotionally demanding research: A proposed conceptual framework	Unspecified	2 EdD students (Doctor of Education (EdD) is a doctoral degree that has a research focus on education)	Qualitative

### Description of the findings

Of the reviewed articles, one was published before the year 2000, while the remaining 16 were published after 2010, reflecting growing interest in the field and possibly an increasing occurrence of problems in this occupational group. Most articles (11) were published in education or educational research journals. The remaining six articles were in the fields of psychology, general and internal medicine, management, health (i.e., public, environmental, and occupational), social sciences, and information science.

Data for six of the 17 studies were collected in the USA and/or Canada, another nine had data from Europe, one had data from Asia and one from an unspecified location (Kumar & Cavallaro, 2018). Of those 17 studies, four focused explicitly on female doctoral students and one on racio–ethnicity.

### Concepts and measurements of well-being used in the studies

The authors of the studies included in the review conceptualized well-being in several different ways. Well-being or lack of it, is typically related to stress, loneliness, psychological distress, depression, and/or social support when viewed through a social/behavioural lens. Yet another perspective on well-being is more clinically based, stressing illness and physical conditions (Cotten, 2008). The studies reviewed here mainly emphasized viewing well-being from a social science perspective, educational research being part of it (Kuper & Kuper, 1996).

Juniper, Walsh, Richardson, and Morley (2012) for example, operationalized the concept of well-being prior to data collection, understanding doctoral student well-being in their quantitative study as “*that part of a researcher’s overall well-being that is primarily influenced by their PhD role and can be influenced by university-based interventions*” (p. 565). This definition is a modification of Juniper’s previous clinical work on the health-related quality of life (HRQL) of persons suffering from asthma (Juniper, 2005). HRQL, which is understood as a sense of well-being, should include good health, a secure social and occupational environment, financial security, spirituality, self-confidence, and strong, supportive relationships (Juniper, 2005). Juniper et al.’s definition of doctoral student well-being was thus derived from her previous definition of well-being as *“that part of a patient’s overall well-being that is primarily determined by health and which can be influenced by healthcare interventions”* (Juniper et al., 2012, p. 564; Juniper, 2005). In both the clinical and non-clinical work, Juniper stresses the subjective experience of well-being and that all aspects of day to day functional life ought to be taken into consideration (Juniper et al., 2012; Juniper, 2005). Juniper assesses how work impacts on doctoral student well-being whereas her previous research investigated how disease impacts patient well-being. Based on the previous clinical work, Juniper et al. (2012) developed and evaluated a questionnaire that ultimately consisted of seven domains: development, facilities, home and health, research, social, supervisor, and university impacting on doctoral student well-being.

Pychyl and Little (1998) applied a concept of subjective well-being (SWB) which was operationalized in the quantitative part of their study using the Composite Affect Scale developed by Diener, Emmons, Larsen, and Griffin (1985), the Satisfaction with Life Scale by Diener et al. (1985), and domain-specific measures of subjective well-being by Palys and Little assessing life satisfaction in seven specific domains (Palys & Little, 1983). Diener views SWB as the person’s evaluation of his or her life (Diener, Napa Scollon, & Lucas, 2003), and uses SWB as the scientific term for happiness and life satisfaction (Edward Diener, 2018). He defines SWB in terms of two separate feelings, positive and negative affect (i.e., the presence of positive emotions and moods, and the absence of unpleasant affect), and satisfaction (e.g., with life, marriage or work) (Diener et al., 2003; Diener, Sapyta, & Suh, 1998).

Another scale developed by Diener et al. (2010) was used by Zahniser, Rupert, and Dorociak (Edward Diener, 2018). This Flourishing scale—previously referred to as the Psychological Well-being Scale—measures socio-psychological prosperity, focusing on social relationships—which are viewed as a complement of SWB. The term “flourishing” is understood to mean the presence of mental health, which according to Keyes is synonymous with SWB (Keyes, 2002) whereas Ryff and Singer (2000) developed a lifespan theory of human flourishing, understanding well-being as *“the striving for perfection that represents the realization of one’s true potential”* (Ryff, 1995, p. 100).

Stubb et al. (2011) explored the concept of experienced socio-psychological well-being, referring to *“doctoral students’ experience of their well-being in their scholarly community”* (p. 35), by asking an open-ended question about the PhD student’s role in that community (see Table II). They did however, similarly to other authors included in this review, adapt a version of the questionnaire NORD MED (Medical Education in Nordic Countries), which was developed for medical students, measuring different theoretical constructs, including motivation, learning and experiences of well-being (Lonka et al., 2008). Although well-being was not defined in the original article presenting NORD MED, it was measured by a total of 13 items, including questions regarding stress, exhaustion, lack of regulation, anxiety, and lack of interest (based on Elo, Leppänen, & Jahkola, 2003; Mäkinen, Olkinuora, & Lonka, 2004; Maslach & Jackson, 1981; Vermunt & Van Rijswijk, 1988). In the articles included in this review, 10 items were used to investigate doctoral student well-being, including one item question on stress, four item questions on exhaustion, two item questions on anxiety (the question “*I (often) have to force myself to work on my thesis”* was reported to belong to different constructs such as a lack of interest scale, anxiety scale and cynicism scale depending on which of the three articles it was used in), and three item questions on lack of interest (Anttila, Lindblom-Ylänne, Lonka, & Pyhältö, 2015; Pyhältö & Keskinen, 2012; Stubb, Pyhältö, & Lonka, 2012). Yet another study investigated experienced well-being in terms of stress, exhaustion, and cynicism in PhD studies (Cornér, Löfström, & Pyhältö, 2017). Even though all questions used resemble the same questions used in modified NORD MED, this article refers to a Doctoral Experience Survey, which leads to the assumption that this may be yet another development of the modified NORD MED. Despite the more or less identical exploration of well-being in the above mentioned four articles, it is referred to as experienced socio-psychological well-being (Pyhältö & Keskinen, 2012), experienced well-being (Anttila et al., 2015; Stubb et al., 2012), and lack of well-being, i.e., burnout (Cornér et al., 2017). Yet, another perspective on experienced well-being may be *Agency well-being*, which is an adaptation of Sen’s capability approach based on the dimensions of agency, well-being, freedom and achievement (Sen, 1993) and can be understood as *“as the success that individuals are having in the pursuit of their core personal projects”* (Pychyl & Little, 1998, p. 458), i.e., paying particular attention to the assessment of individual goals.

Caesens, Stinglhamber, and Luypaert (2014) used measures of perceived stress, job satisfaction, and sleeping problems to investigate well-being, while Hunter and Devine (2016) referred to the concept of emotional well-being without defining it, but clearly stating that they were attempting to understand it by examining emotional exhaustion as measured by the emotional exhaustion scale (Maslach & Jackson, 1981).

The qualitative study by Schmidt and Umans (Schmidt & Umans, 2014) tried to conceptualize doctoral student well-being using the metaphor of *“white-water rafting”* (Schmidt & Umans, 2014, p. 10), seeing it as “*cramped in the interaction between self and structural forces*" (p. 10), i.e., created through interaction between the self (“agent”) and the external factors (“structures”), which the doctoral student is a subject to. In another study, (Haynes et al., 2012) well-being was defined in terms of constitution, force, machine, measurement, and direction, concluding, similar to Schmidt and Umans (2014), that perceived well-being is an individual and social process that is constantly evolving and unique. The operationalization and conceptualization of well-being in the articles reviewed can be found in Table III.

**Table III.** Examples of the operationalization and conceptualization of well-being from the articles included in the review.

Operationalization (mainly quantitative studies)		Article
Experienced well-being	Modified MED NORD 10 items (including stress, exhaustion, anxiety, and lack of interest) Modified MED NORD 10 items (including stress, exhaustion, anxiety, and lack of interest)	Stubb et al., 2012 Anttila et al., 2015
Subjective well-being	Composite Affect Scale, Satisfaction with Life Scale, and domain-specific measures of subjective well-being	Pychyl & Little, 1998
Well-being	58-item questionnaire covering development, facilities, home and health, research, social, supervisor, and university	Juniper et al., 2012
Lack of well-being, such as burnout	Modified Doctoral Experience Survey eight items (including stress, exhaustion and cynicism)	Cornér et al., 2017
Well-being	Job satisfaction, perceived stress scale, and sleeping problems	Caesens et al., 2014
Experienced socio-psychological well-being	“How do you see your own role as a PhD student in your scholarly community?” Modified MED NORD 10 items (including stress, exhaustion, anxiety, and lack of interest)	Stubb et al., 2011 Pyhältö & Keskinen, 2012
Emotional well-being	Emotional exhaustion scale	Hunter & Devine, 2016
Overall well-being	Clinical Psychology Doctoral Student Self-Care Survey including eight item Flourishing Scale	Zahniser, Rupert, & Dorociak, 2017
Psychological well-being	Quality in PhD Processes Questionnaire including seven items on loneliness, insecurity, and exhaustion	Herrmann & Wichmann-Hansen, 2017
Spiritual well-being	20-item spiritual well-being scale consisting of religious and existential well-being dimension	Ziapour et al., 2017
**Conceptualization (mainly qualitative studies)**		
Overall well-being	Shaped by a) academic mask, b) private self, c) other selves, d) protection of self, and e) disadvantages of the academic mask	Shavers & Moore, 2014
Perceptions and experiences of well-being	Interaction between external and individual factors comprise the experiences of well-being, like “white-water rafting”	Schmidt & Umans, 2014
Perceived well-being	An individual and social process that is constantly evolving and unique	Haynes et al., 2012
Well-being	Needed for maintaining a school-work-life-balance and concerned with managing stress levels, maintaining mental and physical health, and creating personal time	Martinez et al., 2013
Researcher well-being	Emotionally demanding research depletes researcher well-being	Kumar & Cavallaro, 2018

**Table IV.** Triggers and outcomes of well-being.

	Triggers	Outcome
1*	Time pressure, anxiety	Stress
	Neuroticism	Decreased well-being in terms of negative affect
	Extraversion	Increased well-being in terms of positive affect, satisfaction with life
	Coping (social support, passion)	Increased well-being (and academic satisfaction)
2	Academic community as source of burden (56%)	Decreased socio-psychological well-being in terms of negative feelings (exhaustion, exclusion, hinder of learning, lack of meaningfulness, insecurity)
		Lower well-being in terms of more stress, exhaustion and anxiety, more lack of interest
	Academic community as source of empowerment (44%)	Increased socio-psychological well-being in terms of positive feelings (enthusiasm, inspiration, support, meaningfulness, contribution, belonging, worthiness)
		Higher well-being in terms of less stress, exhaustion and anxiety, less lack of interest
3	1-Constitution (physical and psychological health)	Definition of well-being
	2-Force (power of influence, of outside control)	
	3-Machine (level of functioning in doctoral program)	
	4-Measurement (balance between program and personal life)	
	5-Direction (guide of thought or purpose)	
4	Development, facilities, home & health, research, social, supervisor, university	
5	Active agent of the scholarly community (30%)	Increased experienced socio-psychological well-being in terms of less exhaustion, anxiety and lack of interest
	Passive object of the scholarly community (70%)	Decreased experienced socio-psychological well-being in terms of more exhaustion, anxiety and lack of interest
6	Thesis as process (49%), product (23%) or both (28%)	Increased well-being in terms of less stress, exhaustion and anxiety, less lack of interest (as well as fewer thoughts of drop out) when viewed as process
7		Seeking well-being by managing stress levels, maintaining mental and physical health, and creating personal time
8	Perceived organizational support	Increased well-being in terms of higher work engagement, higher job satisfaction, less perceived stress and less sleep problems
	Gender (men)	Increased well-being in terms of less perceived stress and less sleep problems
	Work engagement	Increased well-being in terms of higher job satisfaction, and less perceived stress
	Workaholism	Decreased well-being in terms of less job satisfaction, more perceived stress and sleep problems
9	1-Being true to oneself	Perceptions and experiences of well-being
	2-Being in the sphere of others (e.g., scholarly community, men, peers, supervisors, family)	
	3-Performing the balancing act (e.g. working student, dual life, in and out of control)	
10	1-Academic mask	Maintenance of overall well-being
	2-The private self	
	3-The other selves	
	4-Protection of self	
	5-Disadvantages of the academic mask	Negative impact on psychological and emotional well-being
11	Receiving enough feedback	Increased well-being in terms of lower level of stress, exhaustion and anxiety, and less lack of interest
	Discontent with atmosphere	Decreased well-being in terms of wore stress, exhaustion, anxiety, and lack of interest
	Experiences of stress, anxiety, exhaustion, and lack of interest	Consideration of drop out
12	Perceived department/faculty support,	Increased well-being in terms of less emotional exhaustion
	Leader member exchange (relationship between doctoral student and supervisor)	
	Supervisory experience	
	Meeting frequency	
	Gender (female) and intention to leave academia	Decreased well-being in terms of more emotional exhaustion
13	High frequency of supervision	Increased well-being in terms of more satisfaction with supervision
	Consider interrupting studies	Increased well-being in terms of more stress
	Less satisfied with supervisory support	Decreased well-being in terms of more stressed, exhaustion and cynicism
	Support from researcher community	Increased well-being in term of less cynicism
	Sense of equal treatment within researcher community	Increased well-being in terms of less exhaustion and cynicism
	Lack of satisfaction with supervision, lack of equality within the researcher community, lower frequency of supervision	Lack of well-being, such as burnout
14	Collegial research environment, loneliness, insecurity, harsh tone, exhaustion, ownership	Impact on psychological well-being
15	Gender, marital status, age, housing, academic term and field of study	Associated with spiritual well-being
16	Self-care: professional support, professional development, life balance, cognitive awareness, and daily balance	Increased personal well-being in terms of less perceived stress, more positive affect, less negative affect, and more flourishing
17	Emotional demanding research	Decreased researcher well-being
	Self-care	Increased well-being

It can be summarized that well-being, as mentioned earlier, is a complex yet well-used concept. It includes both narrow and broad definitions, is interpreted in various ways, and used differently. Furthermore, well-being also often seems to be studied by focusing on the lack of well-being such as stress, burnout and sleep problems.

### Theoretical models used in the studies

Theoretical models were found to be used as a basis for the theoretical or analytical frameworks of the reviewed papers. Several studies used theory as a basis for their frameworks. Hunter and Devine (2016) used the *leader–member exchange* theoretical perspective to understand the supervisor–doctoral student relationship. Caesens et al. (2014) used *self-determination theory* to understand the extrinsic motivation that drives workaholics, applying *Higgins’s regulatory focus theory* to demonstrate the prevention focus of workaholics. The study further explored the *job demands–resources* theoretical model, used to describe how job resources, namely, social support, can constitute a positive motivational process that enhances work engagement. *Conservation of resources* theory was used to understand the relationship between social support and workaholism. The theories used in the paper established a basis for several hypotheses arranged to form a theoretical framework. A conceptual framework was also proposed by Kumar and Cavallaro (2018) explaining how intertwined the researcher, i.e., the doctoral student, and the research itself are, and how the research process depletes well-being. Pychyl and Little (1998) and Stubb et al. (2012) used the *social ecological model* and the *broaden-and-build theory of emotions*, respectively, to explain the antecedents of doctoral student well-being.

Juniper et al. (2012) applied *impact analysis*—which was previously used to assess well-being in a clinical setting by developing a questionnaire—as a methodological framework when developing a questionnaire for doctoral students covering seven domains (such as home & health, or supervisor), which comprise their well-being.

In the article by Pyhältö and Keskinen (2012), the concept of *relational agency* developed by Edwards (2005) was applied to understand the capacity of doctoral students to work with others to resolve problems by identifying motives, interpreting them and adapting their responses accordingly. The concept is closely related to agency theory, which is mainly used in business studies (Eisenhardt, 1989). Agency theory tries to explain relationships between principals and agents, and to show how problems can be resolved by aligning each other’s goals and interests by means of different incentives. However, while relational agency focuses on the interactive nature of the relationship between principle and agent, agency theory also includes non-relational aspects such as opportunistic behaviour, asymmetry of information and risk aspects (Smith, Umans, & Thomasson, 2018). However, another article refers to agency in terms of “agency well-being” (Little, 2012) when discussing the results of the study.

Another study explicitly stated that they applied theory in discussing their results, namely, *Giddens’ structuration theory* (Schmidt & Umans, 2014) which was used when analyzing the emergence of doctoral student well-being in the interaction between the agent and structure. In addition, other studies used black feminist thought (Shavers & Moore, 2014), phenomenological hermeneutics (Schmidt & Umans, 2014), constructivism (Haynes et al., 2012), the interpretivist paradigm (Martinez et al., 2013), and/or grounded theory (Martinez et al., 2013; Pychyl & Little, 1998) as their overall analytical frameworks. When choosing grounded theory, which two studies did, the ultimate aim was to develop theory based on the findings.

### Methods used in the articles

Of the 17 reviewed studies, eight combined qualitative and quantitative data collection methods, whereas five applied a qualitative and four a quantitative design. Studies combining both qualitative and quantitative data used one or two open-ended questions in questionnaires (Zahniser, Rupert, & Dorociak, 2017; Anttila et al., 2015; Hunter & Devine, 2016; Pyhältö & Keskinen, 2012; Stubb et al., 2011, 2012). The remainder combined questionnaires with interviews/focus groups (Juniper et al., 2012; Pychyl & Little, 1998), which was also chosen as the preferred method among the purely qualitative articles (Haynes et al., 2012; Martinez et al., 2013; Schmidt & Umans, 2014; Shavers & Moore, 2014) with the exception of one article, which applied auto ethnography (Kumar & Cavallaro, 2018).

The qualitative data were analyzed using various analytical tools, such as thematic analysis (Hunter & Devine, 2016), the interpretive perspective (Shavers & Moore, 2014), the lifeworld concept (Schmidt & Umans, 2014), abductive or thematic content analysis (Anttila et al., 2015; Hunter & Devine, 2016; Pyhältö & Keskinen, 2012; Stubb et al., 2011, 2012), grounded theory (Martinez et al., 2013; Pychyl & Little, 1998), metaphorical analysis (Haynes et al., 2012), constant comparative method (Martinez et al., 2013) and retrospective analysis of own experiences (Kumar & Cavallaro, 2018). Quantitative data was analyzed by applying descriptive or comparative statistical methods (Herrmann & Wichmann-Hansen, 2017; Pychyl & Little, 1998; Pyhältö & Keskinen, 2012; Stubb et al., 2011, 2012) as well as variance analysis such as ANOVA (Hunter & Devine, 2016; Juniper et al., 2012; Stubb et al., 2012; Ziapour, Khatony, Jafari, & Kianipour, 2017), correlation (Pychyl & Little, 1998; Stubb et al., 2012), and regression (Hunter & Devine, 2016; Pychyl & Little, 1998).

### Results of the articles

Several articles described doctoral student well-being as related to terms such as self, agent, being true to oneself (Schmidt & Umans, 2014), an individual process (Haynes et al., 2012), time for self (Martinez et al., 2013) or self-care (Zahniser, et al., 2017; Kumar & Cavallaro, 2018), the private self and protection of self (Shavers & Moore, 2014), and internal reflection or an intuitive process focusing on the individual (Haynes et al., 2012), often resulting in various internal battles. These battles or struggles manifest themselves in terms of role conflicts (Pychyl & Little, 1998) or internal conflicts (Martinez et al., 2013), conflicting responsibilities and priorities (Martinez et al., 2013), trade-offs (Martinez et al., 2013), or conflicting goals (Haynes et al., 2012).

Also the meaning doctoral students attribute to their PhD education,—viewing it as a process or a product or both—affects well-being and has been shown to vary among academic disciplines (Stubb et al., 2012). In line with those results, Shavers and Moore (2014) found that overemphasizing academic growth at the expense of emotional and personal development will lead to a lack of wholeness and centredness. Several studies also reported high frequencies of doctoral students considering interrupting their studies. In one study, 56% considered dropping out at some point during the PhD process, and that decision was influenced by experiences of stress, anxiety, exhaustion, and lack of interest (Anttila et al., 2015). Yet another study reported that 43% of the sample considered interrupting their studies (Stubb et al., 2011). Experiences of burnout increased the risk of dropping out, while receiving supervision from several supervisors decreased this risk (Cornér et al., 2017). The notion of doctoral students’ intending to leave academia after completion of the PhD was supported by the study by Hunter and Devine (Hunter & Devine, 2016). About one third of the sample intended leaving academia, which correlated with experiences of well-being in terms of emotional exhaustion during the PhD process. Intention to leave academia after completion of the thesis was higher among students belonging to the hard applied and soft applied disciplines (Hunter & Devine, 2016). Variation in well-being were also found to be related to work condition, i.e., full-time students and those partially belonging to a research group reported higher levels of well-being (Stubb et al., 2011).

Furthermore, one study identified personality traits as having an impact on doctoral student well-being. Pychyl and Little (1998) demonstrated that neuroticism correlated positively with a negative affect, and extraversion with a positive affect. Pychyl and Little (1998) further identified feelings of guilt and anxiety as contributors to stress. The existence of feelings of guilt and frustration was reaffirmed by the study of Schmidt and Umans (2014).

Coping ability is yet another central aspect of doctoral student well-being. For these students, coping mechanisms are necessary to manage stress and to maintain sanity, physical health, and mental well-being—that is, to remain healthy (Martinez et al., 2013). People can respond to stressors in many different ways, for example, working to solve the problem (i.e., problem-focused coping) or reaching out for social support (Carver & Connor-Smith, 2010; Lazarus & Folkman, 1984). The results of the review indicate a strong emphasis on social support as a way of coping.

Crying, isolation, and social interactions with friends all served as coping strategies for the studied doctoral students (Martinez et al., 2013). One study identified *“cling[ing] to the spiritual realm”* (p. 9) as a coping strategy and found that success in developing coping strategies conferred a certain sense of control (Haynes et al., 2012). In addition, planning (i.e., problem-focused coping), and exercise (Martinez et al., 2013) were mentioned as coping mechanisms specific to doctoral students.

Shavers and Moore (2014) found that doctoral students used coping strategies to overcome oppression and to help them persevere academically. An identified coping strategy involved shifting between different selves and using an academic mask; yet, instead of maintaining well-being and fostering optimal, healthy coping, this strategy was categorized as survival-oriented, and using it led to feelings of incompleteness, disconnectedness, and exhaustion. Peer relationships (Schmidt & Umans, 2014), passion, and social support (Pychyl & Little, 1998) were other identified coping resources used by doctoral students.

Yet another strategy with a particular focus on health prevention was mentioned. Self-care according to the Professional Self-care Scale for Psychologists by Dorociak comprises professional support, cognitive awareness, professional development, life balance, and daily balance. All these aspects have been shown to increase well-being, however the first two aspects are of particular importance (Zahniser, et al., 2017). Another study reported self-reflection, yoga, social network support, biking or walking, and compartmentalization as examples of self-care strategies (Kumar & Cavallaro, 2018), stressing in their conceptual framework that individual driven self-care and promotion of self-care by the institution are of equal importance.

Several articles described doctoral student well-being as related to structural forces (Schmidt & Umans, 2014), outside forces (Haynes et al., 2012), external reflection, and social factors (Haynes et al., 2012) as well as being in the sphere of others (Schmidt & Umans, 2014). It consists of personal and academic social interactions, for example, with the spouse and family (Martinez et al., 2013; Schmidt & Umans, 2014), supervisors (Caesens et al., 2014; Cornér et al., 2017; Hunter & Devine, 2016; B. Juniper et al., 2012; Schmidt & Umans, 2014), faculty and the university at large (Caesens et al., 2014; Hunter & Devine, 2016; Juniper et al., 2012; Martinez et al., 2013; Schmidt & Umans, 2014; Zahniser, et al., 2017), and the scholarly community (Cornér et al., 2017; Hunter & Devine, 2016; Schmidt & Umans, 2014; Stubb et al., 2011). Such interactions also relate to social support in general (Juniper et al., 2012; Kumar & Cavallaro, 2018; Martinez et al., 2013; Pychyl & Little, 1998). Attention was mainly paid to the social processes created by interacting with external actors. Finally, one study found that organizational support and supervisor support were positively related to work engagement (Caesens et al., 2014), which in turn had positive effects on well-being, illustrating once again the complexity of the concepts involved.

Several circumstances mentioned in the studies can be summarized as stressors, some of them chronic. Deadlines, limited finances, time, family issues, and relationships were all mentioned as stressors. Another stressor was the need to take on additional responsibilities to position oneself after graduation, while competing commitments led to less enjoyment, motivation issues, problems finishing the dissertation, and ambiguity (Martinez et al., 2013). Managing stress was described as a balancing act, in which the high expectations of various actors, and domestic demands when living a dual life (i.e., being a “superwoman”) had to be balanced to keep stress manageable (Schmidt & Umans, 2014). Lack of control (Haynes et al., 2012; Schmidt & Umans, 2014) was yet another stressor affecting doctoral students’ work, well-being, and health. Pychyl and Little (1998) identified time pressure, time conflicts, and procrastination as stressors.

Some factors could be attributed a dual function: for example, relationships, supervisors, and the scholarly community could all provide support and function as coping mechanisms at times, yet at other times could also be seen as stressors. Examples of how doctoral students' well-being can be influenced and understood is shown in Table IV.

The interaction between the self and external forces is where one’s unique well-being constantly evolves (Haynes et al., 2012; Schmidt & Umans, 2014; Stubb et al., 2011). Yet, when influenced by external forces, well-being can rapidly develop into an upward or downward spiral. If the work–life balance (Zahniser, et al., 2017; Haynes et al., 2012; Martinez et al., 2013; Pychyl & Little, 1998) cannot be maintained, this will ultimately affect the doctoral students’ well-being and produce spill-over effects on their lives more generally.

## Discussion

Inspired by Jackson, Joshi, and Erhardt (2003), the reviewed studies were subjected to a SWOT analysis, identifying the strengths and weaknesses of the research as well as the opportunities and threats. The analysis will be used as a basis for suggestions for future research (Schmidt, 2018).

### Strengths

One strength of the reviewed studies is that most were published after 2010, providing a rather recent view of the situation of doctoral students. Another strength is the number of suggestions made and practical implications identified. Despite Golde’s (2005) comment that research has failed to address how doctoral education could be improved, almost all the reviewed studies attempted to apply their findings, for example, by developing optimal resistance strategies to enhance well-being, such as teaching doctoral students to affirm themselves daily and develop positive thinking patterns (Shavers & Moore, 2014); evaluating and/or developing policies addressing, for example, academic climate or discrimination in PhD programmes (Schmidt & Umans, 2014; Shavers & Moore, 2014); creating an arena for shared meaning using supervisory contracts (Stubb et al., 2012); fostering peer groups as important and meaningful communities for students (Stubb et al., 2011); organizing health and wellness biofeedback labs, recreational sports groups and fitness classes, and seminars on time management (Haynes et al., 2012); training supervisors in mentoring and supervision, and creating a structured model to help advisers provide feedback, in terms of both academic research and relationship management (Hunter & Devine, 2016).

Another strength is the wide sample variation of the articles included (ranging from 2 to 1.760 doctoral students), applying a various number of methodological approaches and study designs. Also, the diverse PhD student body is explored by the inclusion of various academic disciplines such as biology, business administration, health sciences, nursing, informatics, and public health (Schmidt & Umans, 2014), humanities, medicine and behavioural sciences (Pyhältö & Keskinen, 2012; Stubb et al., 2011, 2012), humanities and theology, natural sciences and engineering, social sciences and law, behavioural sciences, economics, and medicine (Cornér et al., 2017), education, chemistry, and agriculture (Hunter & Devine, 2016), art and social sciences (Pychyl & Little, 1998), and psychology (Zahniser, et al., 2017). However, it should be acknowledged that only a few studies address potential differences arising from this diversity.

### Weaknesses

One weakness of the reviewed literature concerns the problematic matter of defining “well-being”, which may create confusion by referring to different concepts, such as emotional well-being, subjective well-being, psychological well-being, socio-psychological well-being, and agency well-being. Well-being is also operationalized in different ways in the various studies and correlated with various other social or health-related concepts such as social support, work engagement, or personality traits, i.e., well-being is used as an input measure, output measure, mediator, and moderator, making it difficult to discern clear causal relationships. Instead, the intertwined relationships create a spider’s web of interactions between all the elements, indicating the complex nature of doctoral student well-being. Most of the studies are inconsistent when it comes to the use of well-being and health concepts, which at times have similar meanings. While for some, well-being is viewed as a central component of health (Martinez et al., 2013), including the World Health Organization’s frequently used definition of 1948 (WHO, 1948), for others, well-being is defined as something greater than health (Galvin & Todres, 2011; Todres & Galvin, 2010). Thus, well-being can be understood as a source of health, and vice versa. The review further showed that meaning and meaningfulness are central attributes of doctoral students’ well-being, as shown by Stubb et al. (2012) and Pychyl and Little (1998). Yet another recurrent aspect in the definitions used is the component of social network/support that serves an important function in PhD student life. Overall, the results of the review resonate well with Ryff’s (1989) holistic definition which highlights the importance of positive relationships with others, personal mastery, autonomy, a feeling of purpose and meaning in life, as well as personal growth and development.

However, some divergence within the definitions remains. For example, Ziapour et al. (Paloutzian & Park, 2015; Ziapour et al., 2017) view existential well-being as being part of spiritual well-being, which emphasizes the sense of life purpose and life satisfaction (Ellison, 1983). Yet the same term is given another meaning by others (Dahlberg, Todres, & Galvin, 2009; Todres & Galvin, 2010). Todres and Galvin define their existential view of well-being (also referred to as the existential theory of well-being) as well-being as a whole before it is structured or categorized into different domains, and refer to well-being as an essential experience that makes all other kinds of well-being possible in its various forms. Cohen, Mount, Tomas, and Mount (1996) on the other hand, include existential well-being in their quality of life scale, implying that—in a clinical setting—it is of more importance for patients with a life-threatening illness. Yalom (1980), who includes death, freedom, isolation and meaning in the existential domain, Cohen et al. (1996) measure existential well-being by asking six questions (such as whether they have achieved their life goals or how they feel about themselves).

Yet another weakness is that most reviewed studies collected their data in Europe, the USA, and Canada, omitting the perspective of the developing countries.

### Opportunities

Much of the reviewed research into doctoral student well-being was conducted in the field of education. One way of further developing the research field would be to expand it to include fields such as psychology, the social sciences, management, and the caring sciences. Theories and models from these fields could help improve our understanding of the complexity of doctoral students’ situations, experiences, and use of suitable coping strategies. They might also improve our understanding of these students’ needs, which, if they are met, would improve their education experience, well-being, and future success and engagement in academia.

Another opportunity would be to use various methods to study all PhD programmes in order to evaluate satisfaction and quality levels from the doctoral student’s perspective. Today, most emphasis is on the *academi*c progress of the student, measured in numbers of publications or conference appearances. Monitoring not only academic progress but also doctoral students’ well-being could lead to changes at the systemic, institutional level. If doctoral students experience a lack of well-being and cannot maintain a healthy work–life balance during the lengthy period of their PhD studies, and might even consider dropping out, this represents a loss for everyone involved. Related to this attrition are economic costs (i.e., waste of departmental, institutional, state, and personal resources), psychosocial costs (i.e., social and emotional costs to students and faculty from loss of invested time and effort and impaired productivity in research projects) (Golde, 2005), and opportunity costs to both the doctoral student and the PhD funder.

Well-developed strategies such as social or problem-focused coping have been shown to be effective for people experiencing stress (Carver & Connor-Smith, 2010). One opportunity to advance research in this area would be to investigate how those strategies could be applied by doctoral students, possibly leading to enhanced well-being. Because this review has found that social support as a coping strategy has been greatly emphasized, more research attention should be paid to problem-focused coping.

Majority of the papers reviewed appear to adopt the hedonic perspective of well- being (e.g., subjective well-being) which provides a potential focus of the eudemonic perspective (e.g., psychological well-being) or combination of both for future research.

Finally, a further possible opportunity could be a focus on gender and other socio-demographic diversities. The reviewed studies are dominated by the experiences of female PhD students, with some of the studies only accounting for women (Haynes et al., 2012; Kumar & Cavallaro, 2018; Schmidt & Umans, 2014; Shavers & Moore, 2014). In countries such as Sweden, the distribution of gender among PhD students is rather even (46% women) but there are vast differences depending on academic discipline, with the greatest variation in technology and agriculture, where female doctoral students account for 27% and 60% respectively (Statistics Sweden, 2016). In countries such as the USA, women account for 44% of the PhDs awarded (Monroe, Ozyurt, Wrigley, & Alexander, 2008) while in Finland 66% of the PhD students in humanities are female, 76% in behavioural sciences, and 71% in medicine (Stubb et al., 2011). These numbers show differences between countries as well as between academic disciplines. As men and women may react and respond differently to triggers such as supervisor support, loneliness and stress, it could be important to give equal attention to both genders.

### Threats

The reviewed studies considered many academic disciplines, such as the humanities, medicine, engineering, and law, all of which apply different paradigms and have different research traditions. The experiences of doctoral students vary widely from discipline to discipline (Golde & Dore, 2001). This makes comparing PhD programmes difficult because they differ in many ways, for example, in course requirements, supervisor involvement, and teaching assignments. Stubb et al. (2012) reported that the experienced meaning of PhD studies as well as the reasons for interrupting studies differed between faculties. Although a national-level review including all disciplines might be advisable to eliminate discrepancies in the quality of doctoral research, programmes, and student well-being, it is believed that harmonizing PhD programmes within disciplines, within countries, or worldwide would not necessarily enhance doctoral student well-being.

## Conclusion

Well-being is a multifaceted concept and a single generally accepted definition of well-being is lacking (Seedhouse, 1995). It is therefore not surprising, although rather problematic, that well-being is described in such different ways in the reviewed studies. There also seems to be confusion in the occupational health field, where well-being is subdivided to the workplace, the social environment and economics, when a more comprehensive approach would be more valuable. Doctoral student well-being might be multidimensional and not limited to a particular setting or role; instead, the present results clearly indicate that it should be studied more comprehensively.

Shavers and Moore (2014) concluded in their article that well-being and academic perseverance cannot coexist simultaneously. Though this review revealed that doctoral students face multiple challenges, it also identified a need for increased awareness of the basic nature of research as a highly challenging endeavour whose progress is unpredictable and nonlinear (Juniper et al., 2012)—as is how doctoral student’s emotions and abilities impact their well-being and PhD work process. HEIs are advised to apply a more student-centred approach when interacting their doctoral students, which could increase the likelihood of these students maintaining their well-being during their PhD studies and, in the long term, maintaining the sustainability of the HEIs.

## Limitations

This review is not without limitations. First, it must be emphasized that the literature review and the interpretation of the findings are subjective in nature. Second, the search was not limited to any context, location, discipline or time frame, it may be incomplete since four databases were used. Yet, the choice of databases was strategical and was reflected upon prior to conducting the search. Third, a limited number of articles were included in the review. However, a systematic search with inclusion criteria that focused on securing a certain level of quality (e.g., peer review, empirics, English) might have decreased the quantity but as the authors carefully read all abstracts/articles found, excluded articles independently, and compared the individual results until agreement was reached, the validity of the review was increased. The exclusion of dissertations was due to the inclusion criteria that required peer review.

Finally, the choice of keywords was elaborated on, and several different writings were included but the searches may not have been exhaustive. Other possible keywords for use in the keyword search, such as job satisfaction, were rejected because job satisfaction only covers work-related factors not aligned with the purpose of this study, which emphasizes well-being as a multifaceted concept rather than singling out components of well-being or certain settings. Because well-being research addresses diverse concepts such as depression, euphoria, global judgments of life satisfaction (Diener et al., 2003) and stress, all negative and positive experiences of well-being are included here to cover as many dimensions as possible of the concept. A key-word search focusing exclusively on the negative aspects, for example, stress, burnout, and exhaustion, was accordingly also rejected.
